# The Practice of Surgery

**Published:** 1854-01

**Authors:** 


					﻿The Practice of Surgery. By James Miller, F. R. S. E., F. Rr
C. S. E., etc., third American from the second Edinburgh edi-
tion, edited by W. T. Surgent, M. D. etc. Philadelphia : Blanch-
ard & Lea, 1854.
The principles of surgery by the same author have been prev-
iously noticed in this Journal. We have no hesitation in recom-
mending this work to the favorable notice of our readers. The
two volumes, although separate treatises, form together a complete
system of surgery, in our opinion the best that has yet been issued
from the English or American press.
In regard to the mechanical execution, it will be sufficient to
state, that it is in the best style of those veteran medical publish-
ers whose imprint it bears.
For sale by D. B. Cooke & Co.. 135, Lake St.	J.
				

